# *Cryptosporidium hominis* Is a Newly Recognized Pathogen in the Arctic Region of Nunavik, Canada: Molecular Characterization of an Outbreak

**DOI:** 10.1371/journal.pntd.0004534

**Published:** 2016-04-08

**Authors:** Karine Thivierge, Asma Iqbal, Brent Dixon, Réjean Dion, Benoît Levesque, Philippe Cantin, Lyne Cédilotte, Momar Ndao, Jean-François Proulx, Cedric P. Yansouni

**Affiliations:** 1 Laboratoire de santé publique du Québec, Institut national de santé publique du Québec, Sainte-Anne-de-Bellevue, Canada; 2 Bureau of Microbial Hazards, Food Directorate, Health Canada, Ottawa, Canada; 3 Direction de la santé environnementale et de la toxicologie, Institut national de santé publique du Québec, Québec City, Canada; 4 Centre d’expertise en analyse environnementale du Québec, Ministère du Développement durable, de l’Environnement, et de la Lutte contre les changements climatiques, Québec City, Canada; 5 J.D. MacLean Centre for Tropical Diseases, McGill University Health Centre, Montreal, Canada; 6 Department of Medical Microbiology, McGill University Health Centre, Montreal, Canada; 7 National Reference Center for Parasitology, Montreal, Canada; 8 Department of Public Health, Nunavik Regional Board of Health and Social Services, Kuujjuaq, Canada; Baylor College of Medicine, UNITED STATES

## Abstract

**Background:**

*Cryptosporidium* is a leading cause of childhood diarrhea in low-resource settings, and has been repeatedly associated with impaired physical and cognitive development. In May 2013, an outbreak of diarrhea caused by *Cryptosporidium hominis* was identified in the Arctic region of Nunavik, Quebec. Human cryptosporidiosis transmission was previously unknown in this region, and very few previous studies have reported it elsewhere in the Arctic. We report clinical, molecular, and epidemiologic details of a multi-village *Cryptosporidium* outbreak in the Canadian Arctic.

**Methodology/Principal Findings:**

We investigated the occurrence of cryptosporidiosis using a descriptive study of cases with onset between April 2013 and April 2014. Cases were defined as Nunavik inhabitants of any age presenting with diarrhea of any duration, in whom *Cryptosporidium* oocysts were detected by stool microscopy in a specialised reference laboratory. *Cryptosporidium* was identified in stool from 51 of 283 individuals. The overall annual incidence rate (IR) was 420 / 100,000 inhabitants. The IR was highest among children aged less than 5 years (1290 /100,000 persons). Genetic subtyping for stool specimens from 14/51 cases was determined by DNA sequence analysis of the 60 kDa glycoprotein (gp60) gene. Sequences aligned with *C*. *hominis* subtype Id in all cases. No common food or water source of infection was identified.

**Conclusions/Significance:**

In this first observed outbreak of human cryptosporidiosis in this Arctic region, the high IR seen is cause for concern about the possible long-term effects on growth and development of children in Inuit communities, who face myriad other challenges such as overcrowding and food-insecurity. The temporal and geographic distribution of cases, as well as the identification of *C*. *hominis* subtype Id, suggest anthroponotic rather than zoonotic transmission. Barriers to timely diagnosis delayed the recognition of human cryptosporidiosis in this remote setting.

## Author Summary

In mid-2013, an outbreak of moderate-to-severe diarrhea caused by *Cryptosporidium* was identified in the Arctic region of Nunavik, Quebec, and it predominantly affected young children. *Cryptosporidium* is a leading cause of childhood diarrhea in low-resource settings, but was previously unknown in this region. This is important because cryptosporidiosis has been repeatedly associated with impaired growth and development, and may interact with other challenges currently faced by children in remote Arctic communities, such as overcrowding and food-insecurity. Although animals in the Arctic have previously been found to harbour *Cryptosporidium parvum*, which can infect both animals and humans, we found that the *Cryptosporidium* identified in the stool of affected people all belonged to the species *C*. *hominis*, which is only known to infect humans. Together with the temporal and geographic distribution of cases, this suggests that cryptosporidiosis in this outbreak was transmitted person-to-person, rather than acquired from contact with animals. The emergence of *Cryptosporidium* infections in Arctic communities may have public health impacts beyond the occurrence of diarrhea.

## Introduction

*Cryptosporidium* is an apicomplexan parasite that is increasingly recognised among immunocompetent hosts as a leading cause of childhood diarrhea in low-resource settings and of waterborne diarrheal outbreaks in high-income countries.[1, 2] *C*. *hominis*, for which humans are the only natural host, and *C*. *parvum*, which infects bovines, wild animals and humans, account for the majority of human infections.[3] *Cryptosporidium* oocysts are transmitted via the fecal-oral route, including by person-to-person spread, from contaminated food or water, or from contact with infected animals. The relative contribution of each mode of transmission to the epidemiology of human disease is incompletely understood, due in part to the fact that traditional diagnostic tools do not differentiate species of *Cryptosporidium*. Symptomatic cases present most often with diarrhea, usually subsiding within 30 days in immunocompetent hosts. However, *Cryptosporidium* infection (with or without diarrhea) disproportionately affects young children at critical stages of growth and brain development, and has been repeatedly associated with reduced linear growth, impaired cognitive development, poor performance at school, less economic productivity, and lower adult height.[2, 4–6] Worryingly, these effects are exacerbated by food scarcity, which is reported by 24–46% of households surveyed in the Canadian Arctic.[7]

In May 2013, the Parasitology Laboratory of the McGill University Health Centre (MUHC) observed the presence of *Cryptosporidium* oocysts in stool specimens from Inuit communities in the Canadian Arctic region of Nunavik, Quebec. Human cryptosporidiosis transmission was previously unknown in this region, and very few previous studies have reported it elsewhere in the Arctic.[8, 9] We report clinical, molecular, and epidemiologic details of a multi-village *Cryptosporidium* outbreak in the Canadian Arctic region of Nunavik, Quebec, starting in 2013.

## Methods

### Setting, case definition, and outbreak investigation

The Nunavik region is located North of the 55^th^ parallel in Quebec, Canada, and comprises a land area of nearly 444,000 km^2^ (171,307.62 sq mi). Approximately 90% of the estimated 12,135 inhabitants are Inuit. We investigated the occurrence of cryptosporidiosis using a descriptive study of cases with onset between 26 April 2013 to 28 April 2014. Cases were defined as Nunavik inhabitants of any age presenting to a health center with diarrhea of any duration, in whom *Cryptosporidium* oocysts were detected by a specialised reference laboratory. Ascertainment of cases was dependent on the decision of people with diarrhea to present for care, and the decision of the healthcare provider to order stool parasitology testing.

In September 2013, public health authorities sent letters to health workers in the region, informing them of an increased incidence of cryptosproridiosis and requesting that unpreserved stool specimens be collected, in addition to the sodium acetate-acetic acid-formalin (SAF)-fixed specimens, from all patients presenting with diarrhea for routine diagnostic testing and molecular analysis of specimens found to harbour *Cryptosporidium* by microscopy. In addition, cases were characterized using a standardised questionnaire including age, sex, village of residence, disease onset date and recovery, clinical features, hospitalization, history of contact with a suspected case of gastroenteritis, and travel during the incubation period.

### Microbiological testing

During the study period, sodium acetate-acetic acid-formalin (SAF)-fixed stool specimens submitted to the McGill University Health Centre (MUHC) Parasitology Laboratory were examined by specialized microscopists using iodine, iron-hematoxylin, and modified acid-fast staining using the Kinyoun Carbol Fuchsin stain. Water quality in the community first-affected (village 1) was assessed by testing water upstream from the municipal treatment plant (12 litres) and treated water from a storage tank (100 litres) for *Cryptosporidium* using EPA Method 1623.

### Molecular epidemiological techniques

Unpreserved stool specimens were requested at the time SAF-preserved specimens were collected, and were stored at -80°C. Species and genotypes of *Cryptosporidium* cases were determined by Nested-PCR amplification and sequencing of a portion of the gene encoding the small subunit (SSU) rRNA, according to Nichols et al.[10] For further genotyping, a 450 bp fragment of the 60 kDa glycoprotein (gp60) gene was amplified according to the protocol described by Iqbal et al.[11] Genetic subtyping was determined by DNA sequence analysis of the 60 kDa glycoprotein (gp60) gene. Phylogenetic analysis of the sequence data of gp60 *C*. *hominis* genotype Id was conducted using the neighbour-joining method. The evolutionary distances were computed using the Kimura 2-Parameter method [12], with *C*. *parvum* (EU164809) as an outgroup.

### Statistical methods

Proportions and rates were calculated using denominators estimated from population projections for 2013 and 2014, Statistics Canada, Institut de la statistique du Québec, and ministère de la Santé et des Services Sociaux du Québec.

## Results

### Case detection and clinical features

From 26 April 2013 to 28 April 2014, 610 SAF-preserved specimens from 283 symptomatic people were submitted from Nunavik for analysis at the McGill University Health Centre. *Cryptosporidium* was identified by stool microscopy using modified acid-fast staining in specimens from 51 of 283 individuals (incidence rate (IR): 4.2 / 1,000 inhabitants [estimated population 12,135]). The median age of people submitting specimens was 34 years (range 1 month– 88 years) and was substantially lower among cases (median 13 years, range 4 months– 65 years). IR were highest among children aged under 5 years (12.9 / 1,000 persons), with a marked preponderance of male cases in children 1 to 4 years (Table 1).

**Table 1:** Number (n), proportion (%) of cases, and incidence rate (IR) of cryptosporidiosis cases per 1,000 inhabitants according to age and sex, Nunavik, April 2013 to April 2014.

Age group (year(s))	Sex		Total n (%)	IR / 1,000 *
	Male	Female		
<1	3	1	4 (7.8)	13.5
1–4	12	3	15 (29.4)	12.7
5–9	1	3	4 (7.8)	2.9
10–19	5	3	8 (15.7)	3.3
20–29	1	4	5 (9.8)	2.4
30–39	3	4	7 (13.7)	4.2
40–59	4	2	6 (11.8)	2.6
≥60	0	2	2 (3.9)	2.6
Total n (%)	29 (56.9)	22 (43.1)	51 (100.0)	4.2
IR / 1,000	4.7	3.7	4.2	

*Denominators were estimated from population projections for 2013 and 2014, Statistics Canada, Institut de la statistique du Québec, and Ministère de la Santé et des Services Sociaux du Québec.

**Fig 1:**
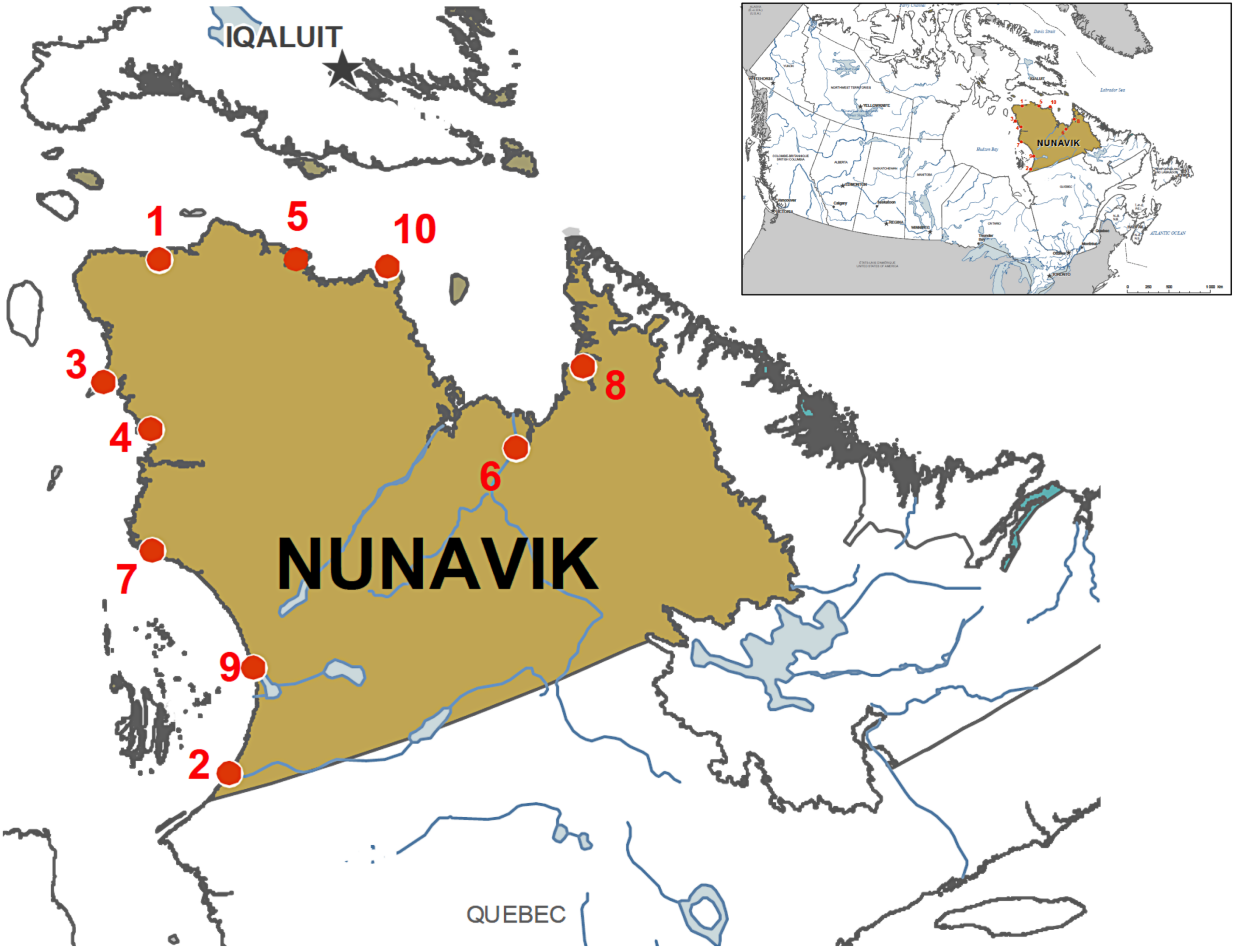
Map of the Nunavik region. Numbers correspond to the geographic location of villages described in the text.

**Fig 2:**
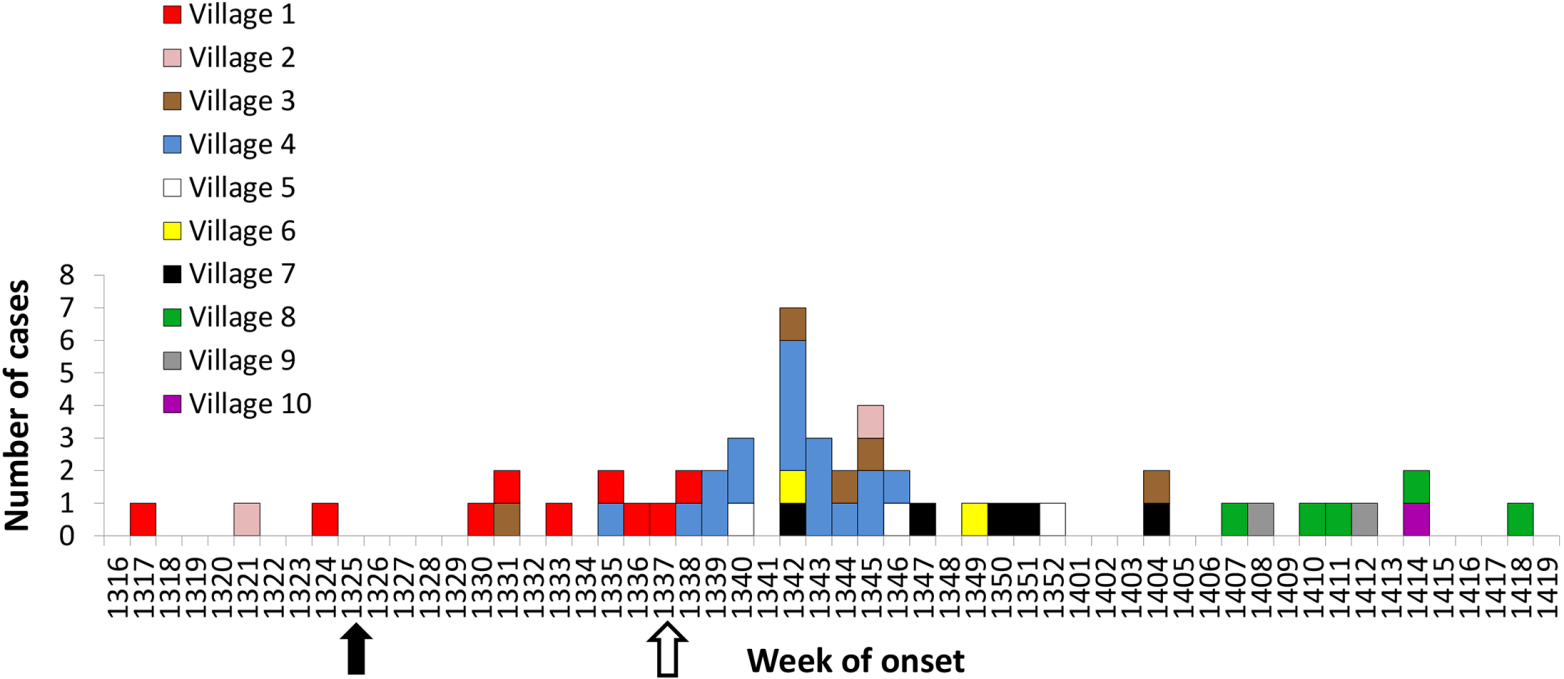
Number of new cases of cryptosporidiosis (n = 51) according to village of residence and CDC week of onset, from April 2013 to April 2014. Week of onset refers to onset of symptoms when known (n = 35) or, if not available, the date of stool specimen collection (available for all cases). The filled arrow denotes the start of routine acid-fast staining for all specimens from Arctic communities; the open arrow denotes the onset of enhanced passive surveillance.

Cases were identified in 10 of the 14 Inuit villages in Nunavik during the study period, each with separate water sources. The IR per village ranged from 0.8 to 9.5 per 1,000 inhabitants. The first cases occurred in Village 1 (Hudson Bay coast, 62°12’N, 75°39’W), from April to September 2013 (Figs 1 and 2). Cases then spread to other villages on the same coast. Village 4 (60.03° N, 77.28° W)—the regional air transport hub on the Hudson Bay coast—accounted for 17 (33%) of the total cases from August to November 2013. Village 8 (58.68° N, 65.95° W) on the coast of Ungava Bay was the last affected, from February to April 2014.

Forty (78%) of the total 51 cases were investigated using a standardised questionnaire (Table 2). The proportion of available information about each variable varied. Seven (24%) of 29 cases were hospitalised. Contact with a person who experienced diarrhea was reported in 17/30 cases (57%). Only 3/20 (13%) cases reported travel outside the village of residence.

**Table 2:** Clinical features of cryptosporidiosis cases, Nunavik, April 2013 to April 2014. “n” denotes number of cases with a given clinical feature, “N” the number of cases for which information was available.

Clinical feature	n / N	Proportion (%)
Diarrhea^†^	35 / 35	100.0
Vomiting	19 / 30	63.3
Nausea	14 / 32	43.8
Weight loss^‡^	10 / 24	41.7
Fever^§^	12 / 29	41.4
Abdominal cramps	11 / 29	37.9
Hematochezia	4 / 23	17.4

^†^The median peak number of liquid stools per day was 7, range 3–20 (information available for 21 cases). Diarrhea lasted a median of 12 days, ranging from 3 to 109 days (information available for 34 cases).

^‡^ Median 500g, range 100 g–23 kg

^§^ Self-reported, with or without temperature measurement

Because of prevailing permafrost in Nunavik, which varies from continuous in the majority of villages to discontinuous in more southerly communities[13], municipal water treatment consists of ultraviolet irradiation and chlorination of a surface water source, several kilometers upstream from wastewater disposal sites. Treated water is then delivered by truck to households. In village 1, water upstream from the municipal treatment plant (12 litres) and treated water from a storage tank (100 litres) was tested for *Cryptosporidium* using EPA Method 1623, and did not yield detectable oocysts. Testing was performed in December 2013, with no cases reported in the community tested during this time.

**Fig 3:**
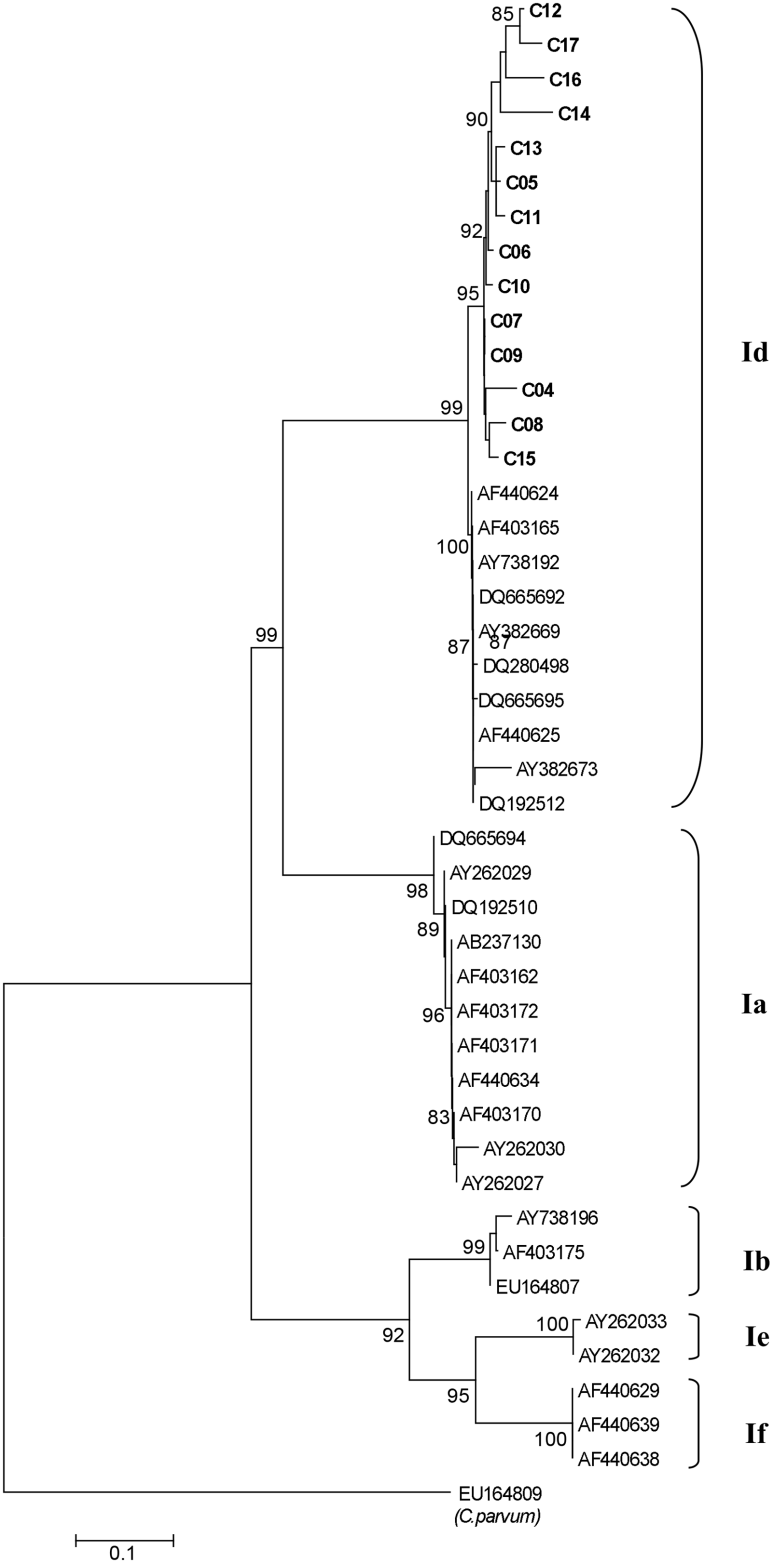
Phylogenetic analysis of *Cryptosporidium hominis* gp60 sequence data using neighbour-joining analysis. Sequences from the present study (C04, C05, C06, C07, C08, C09, C10, C11, C12, C13, C14, C15, C16 and C17) as well as reference sequences representing *C*. *hominis* sub-genotypes (acquired from GenBank) are indicated. The evolutionary distances were computed using the Kimura 2-Parameter method, with C. *parvum* (EU164809) as an outgroup. Bootstrap values greater than 80% from 1,000 replicates are shown.

Finally, for stool specimens from 14/51 cases originating from 6 affected communities, species and genotypes of *Cryptosporidium* were determined by PCR amplification and sequencing of a portion of the gene encoding the small subunit (SSU) rRNA. Genetic subtyping was determined by DNA sequence analysis of the 60 kDa glycoprotein (gp60) gene. BLAST results of these gp60-positive samples showed that all aligned with *C*. *hominis* subtype Id. Phylogenetic analysis of the sequence data of gp60 *C*. *hominis* genotype Id was conducted using the neighbour-joining method (Fig 3). Further analysis demonstrated single isolates of the subtypes IdA13, IdA14G1 IdA14G2R1 and IdA16, and five isolates each of subtypes IdA14 and IdA15. All 14 nucleotide sequences of the gp60 gene of *Cryptosporidium hominis* isolates were deposited in GenBank under accession numbers KU179651 to KU179664 (Table 3).

**Table 3:** GeneBank accession numbers of gp60 gene of *Cryptosporidium hominis* subtypes isolated from patients with diarrhea in the present study.

Species	Host	Genotype	Subgenotype	GenBank accession numbers
*Cryptosporidium hominis* (C04)	Humans	Id	A14	KU179651
*Cryptosporidium hominis* (C05)	Humans	Id	A16	KU179652
*Cryptosporidium hominis* (C06)	Humans	Id	A15	KU179653
*Cryptosporidium hominis* (C07)	Humans	Id	A14	KU179654
*Cryptosporidium hominis* (C08)	Humans	Id	A15	KU179655
*Cryptosporidium hominis* (C09)	Humans	Id	A15	KU179656
*Cryptosporidium hominis* (C10)	Humans	Id	A14	KU179657
*Cryptosporidium hominis* (C11)	Humans	Id	A15	KU179658
*Cryptosporidium hominis* (C12)	Humans	Id	A15	KU179659
*Cryptosporidium hominis* (C13)	Humans	Id	A14	KU179660
*Cryptosporidium hominis* (C14)	Humans	Id	A14G1	KU179661
*Cryptosporidium hominis* (C15)	Humans	Id	A13	KU179662
*Cryptosporidium hominis* (C16)	Humans	Id	A14	KU179663
*Cryptosporidium hominis* (C17)	Humans	Id	A14G2R1	KU179664

## Discussion

The present report details the occurrence of human cryptosporidiosis in the Arctic, in a region where this disease was not previously known. After the study period, the outbreak persisted in Nunavik until the end of 2014, where an additional 18 cases were reported; the annual IR for 2013 and 2014 were respectively 322.5 and 246.4 / 100,000 persons. The annual IR for the rest of the province of Quebec, excluding Nunavik, were respectively 0.5 and 0.8 / 100,000 persons in 2013 and 2014. Cryptosporidiosis is nationally notifiable since 2000; 830 cases were reported in Canada in 2013, the most recent year for which data are available, yielding an IR of 2.4 / 100,000 persons (personal communication; Pushpa Narayanan, Public Health Agency of Canada [2015-07-30]). Thus, the IR of cryptosporidiosis in Nunavik was much higher than for the rest of Quebec and Canada as a whole in 2013 and 2014.

In Nunavik, *Cryptosporidium* spp. has been previously detected in ringed seals, bearded seals and blue mussels, making food-borne transmission through the consumption of marine animals a possible route of infection.[14, 15] Elsewhere in Arctic North America, dogs and caribou have been found to carry *Cryptosporidium* and may be another source of zoonotic infection.[16, 17] However, in this first observed outbreak of human disease in the region, the identification by molecular typing of *C*. *hominis* subtype Id in all 14 specimens tested rules-out zoonotic transmission for these specimens, though the possibility remains that other *Cryptosporidium* subtypes could have been present in specimens that were not characterised. Further analysis demonstrated single isolates of the subtypes IdA13, IdA14G1 IdA14G2R1 and IdA16, and five isolates each of subtypes IdA14 and IdA15. The latter two subtypes, being the most prevalent in the present study, suggest common anthroponotic sources of infection in these patients. It is interesting to note that, globally, *C*. *hominis* subtype Id is found less frequently compared to *C*. *hominis* subtype Ib.[3] Human infections with subtype Id have previously been reported in Ontario[18] and British Columbia[19], but not in Arctic ecosystems. No animal-derived subtype Id strains have been described. To the best of our knowledge, the *C*. *hominis* “Id subtypes” identified in this study have not been reported previously in Canada. In contrast to the current report, the only other study involving the molecular characterization of *Cryptosporidium* infections in humans in the Arctic, reported only *C*. *parvum* IIa in diarrhoeic patients in Nunavut.[9]

The temporal and geographic distribution of cases we observed (Fig 2) further support predominant person-to-person transmission. A common food vehicle or water source would not explain the observed global epidemiologic profile of this outbreak. Finally, microbiological testing of the water supply chain in Village 1 did not detect any parasites or unacceptable coliform counts, though this assessment was limited by the fact that we were only able to test a single village, at a time when no cases were recorded.

In this study, a higher IR was observed in children younger than 5 years of age. This is in keeping with what is known about cryptosporidiosis in low-income countries where *Cryptosporidium* is a leading cause of infectious diarrhea in young children but not in older age groups.[2] Secondary cases among family members are well documented.[20] Multiple factors likely contribute to these observations, including less frequent use of appropriate hand hygiene and immature cellular immunity in this age group.[21]

Protocols in our laboratory did not call for routine acid-fast staining of diarrheal specimens from Arctic communities prior to the current outbreak, raising the possibility that human cryptosporidiosis occurred in Nunavik before 2013. The initial cases were detected because they were of sufficiently heavy burden to be visible to experienced technologists on iodine and iron-hematoxylin stained-specimens. The requirement for special laboratory procedures, lack of local diagnostic capacity in affected communities (up to 1,900 km north of Montreal, Qc), and low sensitivity of microscopy, likely result in underestimated disease burden in this region. Moreover, the public health significance of human cryptosporidiosis may go under-recognised because of a combination of prolonged shedding of oocysts from asymptomatic hosts and the fact that long-term developmental impacts in children are not limited to those with diarrhea.[5]

There are a number of limitations to this study. First, the original source of the *Cryptosporidium* oocysts that caused the outbreak remains unknown. It is possible that seasonal outbreaks have gone unrecognised in the past because of health-seeking behaviours and barriers to appropriate diagnostic testing. Secondly, only laboratory-confirmed cases were included, leading to a likely underestimation of the scope of the outbreak. Thirdly, logistic and geographic obstacles prevented a formal case-control study and the collection of detailed information about possible disease exposures. Available data was limited to questions about contact with a suspected case (before or after disease onset) and travel outside the village of residence, and it proved very difficult to obtain the necessary information from all the respondents. Finally, we were able to perform molecular characterisation of only a subset of outbreak stools because it proved difficult to encourage clinicians and patients to provide additional unpreserved stool specimens for PCR. The latter point proved a key limitation for rapid characterisation of this outbreak and illustrates the need for innovation in point-of-care specimen collection techniques for enteric infections.

In summary, we describe an outbreak of cryptosporidiosis in a region where no *Cryptosporidium* transmission was reported before. In addition, we identified an anthroponotic genospecies of *Cryptosporidium* in the Arctic, with epidemiologic features that suggest sustained person-to-person transmission as is more typically seen in low-income countries than elsewhere in North-America. Finally, the heavy burden of neglected parasitic diseases affecting Inuit communities has been well described [22], and this work adds *Cryptosporidium* to their ranks. The high IR of the Nunavik outbreak and the recent recognition of widespread human cryptosporidiosis in neighbouring Nunavut [23] are cause for considerable concern about the possible long-term effects on growth and development of children in Inuit communities facing myriad other challenges. Though repeated enteric infections are thought to be prevalent in these regions [24], little data currently inform our understanding of their clinical burden and etiologic spectrum. Such data are needed for the development of preventive strategies that take into account human practices and environmental changes that disproportionately affect the Arctic.
